# Immune Checkpoint Inhibition in Acute Myeloid Leukemia and Myelodysplastic Syndromes

**DOI:** 10.3390/cells11142249

**Published:** 2022-07-20

**Authors:** Yasmin Abaza, Amer M. Zeidan

**Affiliations:** 1 Department of Hematology and Oncology, Northwestern University, Robert H. Lurie Comprehensive Cancer Center, Chicago, IL 60611, USA; yasmin.abaza@nm.org; 2 Section of Hematology, Department of Medicine, Smilow Cancer Center, Yale University, New Haven, CT 06511, USA

**Keywords:** acute myeloid leukemia, CD47, immune checkpoint inhibitors, myelodysplastic syndromes, TIM-3

## Abstract

Immune checkpoint inhibitors (ICIs) have revolutionized the treatment of many solid tumors, with limited progress made in the area of myeloid malignancies. The low mutational burden of acute myeloid leukemia (AML) is one potential reason behind the lack of activity of T-cell harnessing ICIs, particularly CTLA-4 and PD-1 inhibitors. Innate immune checkpoints play a critical role in the immune escape of AML and myelodysplastic syndromes (MDS). The CD47 targeting agent, magrolimab, has shown promising activity when combined with azacitidine in early phase trials conducted in AML and higher-risk MDS, especially among patients harboring a *TP53* mutation. Similarly, sabatolimab (an anti-TIM-3 monoclonal antibody) plus hypomethylating agents have shown durable responses in higher-risk MDS and AML in early clinical trials. Randomized trials are currently ongoing to confirm the efficacy of these agents. In this review, we will present the current progress and future directions of immune checkpoint inhibition in AML and MDS.

## 1. Introduction

Allogeneic stem cell transplantation (allo-SCT) represents the most effective immune-based treatment modality, to date, in myeloid neoplasms due to the antileukemic effect of allogeneic grafts mediated mainly by their graft-versus-leukemia (GVL) effect [1]. Long-term outcomes of patients with acute myeloid leukemia (AML) treated with intensive chemotherapy without proceeding with allo-SCT in the first remission are poor, with a 10-year overall survival (OS) rate of less than 20%; emphasizing the importance of allo-SCT in eradicating leukemic cells, thereby inducing durable remissions [2]. The curative potential of allo-SCT in AML and myelodysplastic syndromes (MDS) coupled with the preservation of the T-cell population in the bone marrow of AML patients and increased expression of targetable immune receptors are the main reasons behind the growing interest in using T-cell-harnessing therapies in myeloid neoplasms [3].

Immune checkpoint inhibitors (ICIs) have revolutionized the outcomes of many solid tumors [4,5]. Cytotoxic T-lymphocyte antigen-4 (CTLA-4) and programmed death receptor-1 (PD-1) are the most well-known inhibitory immune checkpoints of the adaptive immune system and were the first receptors to be clinically targeted in cancer therapy [6]. Despite the robust clinical activity of CTLA-4 and PD-1/PD-L1 inhibitors in solid tumors, they have failed to show similar activity in myeloid malignancies [5]. This discrepancy in clinical activity may be attributed to the low mutational burden and DNA mismatch repair proficiency in AML compared to solid malignancies [7,8,9,10].

Recently, there has been increasing recognition of the importance of the innate immune system in the pathogenesis of myeloid neoplasms. CD47 has been identified as a key macrophage checkpoint and a promising target for AML and MDS [11,12]. Magrolimab has shown encouraging clinical activity in AML and higher-risk MDS (HR-MDS) when combined with azacitidine, especially in patients harboring a *TP53* mutation, providing the basis for several ongoing randomized clinical trials [13,14].

**Figure 1:**
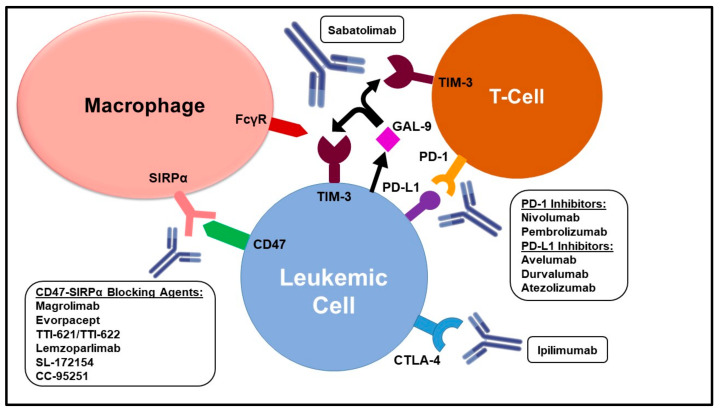
Main Mechanisms of Immune Evasion in Acute Myeloid Leukemia and Myelodysplastic Syndromes.

T-cell immunoglobulin domain and mucin domain-3 (TIM-3), another T-cell immune checkpoint, are being investigated as therapeutic targets in AML and HR-MDS. Their selective expression on the surface of leukemic stem cells (LSC) and association with an immunosuppressive phenotype make them promising targets in myeloid malignancies [15,16]. In this review, we will discuss the main pathways involved in the immune escape of myeloid neoplasms and present the current progress and future directions of immune checkpoint inhibition in AML and MDS (Figure 1 and Table 1). 

Abbreviations: CTLA-4: cytotoxic T-lymphocyte antigen-4; FcγR: fragment crystallizable gamma receptor; GAL-9: galectin-9; PD-1: programmed death receptor-1; PD-L1: programmed death-ligand 1; SIRPα: signal regulatory protein alpha; TIM-3: T-cell immunoglobulin domain and mucin domain-3.

## 2. CTLA-4 Inhibition 

Expressed on the surface of T-cells, CTLA-4 inhibits T-cell maturation and differentiation by outcompeting the T-cell co-stimulatory receptor, CD28, in binding to CD80 and CD86 ligands, thereby counteracting CD28 signaling and hindering T-cell activation [6]. In preclinical studies, CTLA-4 blockade in murine bone marrow chimeras induced potent anti-leukemic activity while avoiding graft-versus-host disease (GVHD), suggesting the potential value of adjuvant CTLA-4 blockade following allo-SCT in AML. In addition, studies have shown increased expression of co-stimulatory molecules, including CD80 and CD86, in AML, which have been associated with high relapse rates and poor prognosis [17,18,19].

### Ipilimumab

Despite the abovementioned findings, single-agent ipilimumab has shown modest clinical activity in both relapsed/refractory (R/R) AML and MDS [20,21]. In a phase 1/1b study assessing ipilimumab in relapsed hematologic malignancies following allo-SCT, complete remission (CR) was achieved in five patients with AML (N = 12, including four patients with extramedullary disease), of which four patients had durable remissions lasting more than 1 year [20]. These results suggest the particular susceptibility of extramedullary disease to ICIs. The main mechanisms behind immune escape after allo-SCT include decreased expression of co-stimulatory molecules and loss of recipient HLA haplotype expression by leukemic cells, in addition to increased expression of checkpoint receptors by donor-derived T-cells [20,22,23]. These changes potentially explain the activity of ipilimumab in the post-SCT setting by enhancing the GVL effect of donor T-cells. Of the four patients with AML and extramedullary disease who responded to ipilimumab, three developed GVHD, supporting this theory [20].

In another phase 1b trial conducted by Zeidan et al., ipilimumab showed limited single-agent activity in patients with HR-MDS who have failed hypomethylating agents (HMAs) [21]. Of the 29 patients treated in this study, 1 patient achieved a marrow CR for an overall response rate (ORR) of 3.4%; the duration of response was 3 months [21]. Seven patients (24%) achieved prolonged stable disease for ≥46 weeks, including three patients with stable disease for more than 1 year. A total of five patients (17%) were successfully bridged to allo-SCT without increased toxicity [21]. 

## 3. PD-1/PDL-1 Blockade

The expression of tumor-specific antigens by cancer cells makes them susceptible to recognition and cytolysis by CD8^+^ T cells [24]. PD-1 is a negative co-receptor expressed by activated T-cells. Interaction of PD-1 with its ligand, programmed death-ligand 1 (PD-L1) expressed by tumor cells, dampens anti-tumor T-cell responses through inducing T-cell apoptosis and attenuating T-cell receptor signaling thus impeding effector T-cell responses [25,26,27]. Interruption of the PD-1/PD-L1 signaling pathway can rescue exhausted T cells and restore anti-tumor responses [28,29]. Therefore, immune checkpoint blockades using anti-PD-1/PD-L1 monoclonal antibodies have emerged as promising novel therapeutic agents. Despite the importance of PD-1/PD-L1 blockade in the treatment of various solid tumors, the role of this pathway in AML and MDS remains largely unknown. 

PD-L1 is minimally expressed by leukemic cells at initial diagnosis, with expression significantly upregulated at the time of disease progression [3,28,30]. PD-L1 has also been found to be overexpressed in patients with *TP53*-mutant AML and MDS, contributing to the immune evasive phenotype of this molecularly defined subgroup of patients [31]. In preclinical studies, PD-L1 blockade augmented anti-tumor T-cell responses resulting in a decrease in leukemic burden and prolonging survival of AML murine models [28]. Pidilizumab (CT-011), humanized IgG1 anti-PD1 monoclonal antibody, was found to be safe and well-tolerated in a phase 1 trial of 17 patients with advanced hematologic malignancies, including 8 patients with AML and MDS [32]. Responses were observed in 33% of patients, with one patient with AML experiencing a reduction in peripheral blood blasts from 50% to 5% [32]. 

Given the very modest single-agent activity of PD-1/PD-L1 inhibitors in AML and MDS, combination regimens using agents that enhance PD-1/PD-L1 expression were investigated [32]. HMAs have been found to upregulate the expression of PD-1, PD-L1, PD-L2, and CTLA4 in patients with AML and MDS, which is thought to be one of the mechanisms of HMA resistance [33]. In addition, HMAs upregulate the expression of tumor antigens and co-stimulatory molecules in cancer cells, supporting their use in combination with PD-1/PD-L1 inhibitors [34,35]. Similarly, cytarabine was found to increase the expression of co-stimulatory molecules by AML cells whilst decreasing the expression of PD-L1, making them more susceptible to cytotoxic lymphocyte-mediated killing [36]. Based on these findings, numerous clinical trials combining PD-1/PD-L1 inhibitors with HMAs and cytotoxic chemotherapy have been recently conducted in AML and MDS in an attempt to reinstate immunosurveillance and improve patient outcomes. 

### 3.1. PD-1 Inhibitors

#### 3.1.1. Nivolumab

In a recent phase 2 study, nivolumab administered in combination with azacitidine was assessed in 70 patients with R/R AML, including 45 patients (65%) with prior exposure to HMA-based therapy [37]. The ORR was 33%, of which 22% of these patients achieved CR (N = 4) and CR with incomplete count recovery (CRi, N = 11) with a median OS of 6.3 months; responses were higher among HMA-naïve patients (ORR rate: 52%) [37]. These results compared favorably to a historical control of 172 patients with R/R AML treated on HMA-based salvage clinical trials (30% had prior HMA therapy) with an ORR of 20% and median OS of 4.6 months (*p* = 0.013) [37]. In line with other studies, grade 3–4 immune-related adverse events (irAEs) were reported in 11% of patients [37,38]. Upregulation of CTLA-4 expression on CD4^+^ and CD8^+^ T cells occurred with therapy among non-responders compared to responders, suggesting CTLA-4 overexpression as a potential mechanism of resistance to PD-1 blockade [37]. 

Due to CTLA-4 upregulation, a second cohort was added to this study in which 31 patients with R/R AML were treated with ipilimumab in combination with azacitidine plus nivolumab in an attempt to further enhance T cell responses [39]. The ORR rate among efficacy evaluable patients (N = 24) was 46% (CR/CRi rate: 36%) with a median OS of 10.5 months, comparing favorably to azacitidine plus nivolumab. As expected, grade 3–4 irAEs were observed in 25% of patients, higher than that seen with azacitidine plus nivolumab [39]. Given these encouraging results, there is an ongoing phase 1 trial assessing nivolumab plus ipilimumab in the treatment of patients with R/R AML and MDS following allo-SCT (NCT03600155) and an additional study assessing nivolumab and/or ipilimumab with or without azacitidine in MDS (NCT02530463).

To assess the activity of nivolumab in the frontline setting, a phase 2 study was conducted combining nivolumab with idarubicin plus cytarabine in patients with newly diagnosed AML (N = 42) and HR-MDS (N = 2) [40]. The composite CR rate was 78%, of which 79% had no evidence of measurable residual disease (MRD) using multiparameter flow cytometry (MFC) [40]. Nineteen patients were bridged to allo-SCT, with thirteen patients (68%) developing GVHD (grade 1–2 in eight and grade 3–4 in five patients) [40]. The combination was well-tolerated without excess irAEs [40]. Median OS for the whole cohort was 18.5 months, and for those who proceeded with allo-SCT was 25 months [40]. Notably, there was no difference in the OS between responders who continued on therapy beyond remission and those bridged to allo-SCT, suggesting the potential ability of nivolumab to restore anti-tumor immune surveillance and eradicate MRD [40]. Subsequently, a phase 2 pilot study assessing nivolumab as maintenance therapy in high-risk AML (N = 15) was conducted [41]. Nivolumab showed a modest ability to eradicate MRD and extend remissions with a median recurrence-free survival of only 8.5 months, providing no support for its use as a single agent in this setting [41].

#### 3.1.2. Pembrolizumab

Pembrolizumab, in combination with azacitidine, was assessed in a multicenter phase 2 study in patients with both newly diagnosed and R/R AML [42]. Of the 37 patients with R/R AML enrolled, 29 patients were evaluable for response with an ORR of 55% [CR/CRi: 14%; partial remission (PR): 4%; hematologic improvement (HI): 14%; stable disease: 24%] [42]. Median OS for the entire cohort was 10.8 months. Among the 22 patients with newly diagnosed AML unfit for intensive chemotherapy, 17 patients were evaluable for response with an ORR of 94%, of which 47% achieved CR/CRi (6 and 2 patients, respectively) [42]. Median OS for this cohort was 13.1 months [42]. The combination was well tolerated and particularly active in the newly diagnosed setting [42]. In another study conducted by Goswami et al., pembrolizumab plus 10 days of decitabine was tested in 10 patients with R/R AML [43]. In line with the previous study, responses were observed in six patients; one achieved a morphologic leukemia-free state, three with stable disease, and two achieved CR [43]. The combination was found to be safe with a median OS of 10 months [43]. 

To assess the impact of pembrolizumab on intensive chemotherapy, Zeidner et al. conducted a phase 2 study of high-dose cytarabine (HiDAC) followed by pembrolizumab in 37 patients with R/R AML [44]. The ORR, CR/CRi rate, and median OS were 46%, 38%, and 11.1 months, respectively, with 50% of the CR/CRi patients achieving MRD negativity [44]. The greatest benefit was observed in patients who received this combination as their first salvage regimen with an ORR and median OS of 54% and 11.3 months, respectively [44]. Nine patients (24%) were bridged to allo-SCT of which six relapsed post-SCT, and two died of infectious complications [44]. In a subsequent retrospective matched cohort analysis, the outcomes of these 9 patients were compared to a historical cohort of 18 patients with AML who underwent allo-SCT without prior exposure to ICIs to evaluate the safety of ICI use prior to allo-SCT [45]. OS was comparable between both cohorts with low rates of treatment-related mortality (1% vs. 17%). Although there was no increase in the risk of acute or chronic GVHD in the pembrolizumab group, seven of nine patients received only one dose of pembrolizumab prior to allo-SCT, which may not have been enough exposure to elicit post-SCT complications [45]. These results are consistent with numerous other studies that have shown the safety of consolidative allo-SCT after ICIs in both AML and MDS, especially with the use of post-transplantation cyclophosphamide prophylaxis [46,47,48]. 

Based on these encouraging results, there are two ongoing randomized trials designed to assess the ability of pembrolizumab to eradicate MRD and therefore prolong OS when combined with intensive chemotherapy (NCT04214249) or with azacitidine plus venetoclax (NCT04284787) in patients with newly diagnosed AML. 

In the phase 1b KEYNOTE-013 study assessing single-agent pembrolizumab in patients with intermediate to high-risk MDS after HMA-failure, five patients (19%) achieved marrow CR, 12 (44%) stable disease, and five (19%) had HI [49]. The study failed to meet its primary endpoint since none of the patients achieved complete or partial remission [49]. Median OS was 6 months, consistent with previous reports [50,51]. Similar results were seen in another phase 2 study combining pembrolizumab with azacitidine in patients with both untreated and HMA-failure intermediate to high-risk MDS [52]. In HMA-naïve patients (N = 17), the ORR was 76%, with a CR rate of 18% and median OS not reached after a median follow-up of 12.8 months; whereas in the HMA-failure cohort (N = 20), the ORR was 25% with a CR rate of 5% and median OS of 5.8 months [52]. Overall, pembrolizumab did not show any survival benefit in patients with HR-MDS after the failure of HMAs.

Notably, the rates of irAEs reported in patients with AML and HR-MDS treated in trials using PD-1/PD-L1 and CTLA-4 immune checkpoint inhibitors are similar to those observed with solid tumors [53,54]. Due to the absence of specific guidelines for the management of irAEs in patients with myeloid malignancies, these adverse events are typically managed as per the published guidelines for solid tumors [53]. The main challenges associated with the assessment and management of irAEs in patients with myeloid neoplasms include: (1) profound thrombocytopenia limiting the ability to perform invasive diagnostic procedures and collect tissue biopsies; (2) difficulty in distinguishing between pneumonitis and pneumonia, the latter is commonly seen in this patient population; (3) severe neutropenia and high rates of infections, particularly fungal infections, hindering the prolonged use of corticosteroids; (4) difficulty in diagnosing hematologic irAEs, such as immune thrombocytopenia and aplastic anemia, due to baseline cytopenias [54]. 

### 3.2. PD-L1 Inhibitors

To date, anti-PD-L1 inhibitors have failed to show clinical activity in AML and MDS. In a single-arm phase 1 trial assessing avelumab in combination with decitabine in previously untreated patients with AML unfit for intensive chemotherapy (N = 7), responses were modest, with one patient achieving CR and three presenting stable disease as the best response to therapy [55]. This CR rate was similar to historical data using single-agent decitabine, suggesting no benefit with the addition of avelumab [56]. Similar results were seen in the international, randomized phase 2 FUSION-AML-001 study comparing azacitidine with or without durvalumab as frontline treatment in older patients with AML unfit for intensive chemotherapy [57]. This study enrolled 129 patients with AML; 64 patients were treated with the combination, and 65 received azacitidine monotherapy [57]. There was no difference in the CR/CRi rate (31% vs. 35%), median OS (13 vs. 14.4 months), and duration of response (24.6 vs. 51.7 weeks) between both arms [57]. 

In the relapsed/refractory setting, a phase 1b/2 study was conducted to assess azacitidine plus avelumab in 19 patients with R/R AML, of which 63% of patients had prior exposure to HMAs [58]. The CR/CRi rate was 10.5% with a median OS of 4.8 months, comparable to the historical CR/CRi rate of 16% achieved with single-agent HMAs [58,59]. Exploratory analysis performed in these studies suggests that overexpression of PD-L2 by AML blasts and monocyte-restricted increase in PD-L1 expression with therapy are potential reasons behind the lack of clinical activity of PD-L1 inhibitors in AML [57,58]. 

In newly diagnosed HR-MDS, the FUSION-AML-001 trial failed to show improvement in patient outcomes with the use of azacitidine plus durvalumab compared to azacitidine alone. Of the 84 patients enrolled (42 in each arm), there was no significant difference in the ORR (61.9% vs. 47.6%, *p* = 0.18) and median OS (11.6 vs. 16.7 months; *p*= 0.74) between both arms [60]. Furthermore, toxicities, including hematologic adverse events and infections, were higher with the combination regimen (89.5% vs. 73.2% and 86.8% vs. 65.9%, respectively) [60]. Similarly, atezolizumab alone and in combination with azacitidine failed to show clinical benefit in patients with R/R or HMA-naïve MDS [61]. Furthermore, atezolizumab plus azacitidine was associated with a high treatment-related death rate in HMA-naïve MDS patients leading to early trial termination [61]. Collectively, these results suggest that there is no clinical benefit from the use of anti-PD-L1 inhibitors in the treatment of AML and MDS.

## 4. CD47-SIRPα Blockade 

CD47 is a dominant macrophage immune checkpoint that malignant cells utilize to evade innate immunity. CD47 relays an anti-phagocytic “don’t eat me” signal upon binding to its receptor signal-regulatory protein alpha (SIRPα) on the surface of macrophages. CD47- SIRPα interaction leads to recruitment of downstream Src homology-2 domain-containing protein tyrosine phosphatases (SHP-1 and SHP-2), preventing the accumulation of myosin-IIA at the phagocytic synapse, thereby inhibiting macrophage-mediated tumor phagocytosis [62]. 

The balance between pro- and anti-phagocytic signals is essential to maintaining cellular homeostasis [63]. Compared to normal hematopoietic stem cells, CD47 is upregulated in AML and HR-MDS, inferring poor prognosis due to evasion of phagocyte-mediated immune surveillance [11,12,64,65,66,67]. Calreticulin, a dominant pro-phagocytic signal, is also overexpressed on the surface of LSC, making it particularly susceptible to CD47 blockade [63]. In vivo and in vitro studies have shown preferential phagocytosis and elimination of AML LSC with anti-CD47 monoclonal antibodies supporting their use in the treatment of AML and HR-MDS [12,68]. Currently, there are numerous agents targeting the CD47-SIRPα axis under investigation in clinical trials (Table 2) [69]. These novel agents are either monoclonal antibodies that directly block CD47 or decoy receptors (SIRPα-IgG Fc domain). 

### 4.1. Magrolimab (Hu5F9-G4)

Magrolimab, first-in-class humanized anti-CD47 antibody, potently induced macrophage-mediated phagocytosis of human AML cells in vitro and in vivo, thereby eradicating leukemic cells and inducing durable remissions in patient-derived xenograft (PDX) mouse models [68]. In the phase 1 CAMELLIA trial conducted on 15 patients with R/R AML, magrolimab was well tolerated and showed modest single-agent activity, with 73% of patients achieving stable disease and 40% experiencing reductions in bone marrow blast count (mean decrease of 27%, range 5–67%) [70]. In an attempt to enhance the antileukemic activity of CD47 blockade, combination regimens using agents that augment the expression of prophagocytic signals on leukemic cells, therefore synergistically inducing leukemic phagocytosis, were developed [71].

In preclinical studies, azacitidine was found to simultaneously upregulate cell surface expression of both CD47 and calreticulin in AML and MDS cell lines [72,73]. In vitro, azacitidine plus magrolimab synergistically increased macrophage-mediated phagocytosis of AML cells which translated into a significant improvement in the long-term survival of AML xenograft mouse models [71]. Based on these results, a phase 1b trial was conducted to assess the efficacy and safety of magrolimab in combination with azacitidine in 91 patients with previously untreated AML unfit for intensive chemotherapy (N = 52) and intermediate to very high-risk MDS (N = 39) [13,14]. In efficacy evaluable MDS patients (N = 33), the ORR was 91%, with the majority of responses consisting of CR (42%) and marrow CR (24%), of which 22% were MRD negative by MFC [14]. The median time to response was 1.9 months, faster than that achieved with azacitidine alone [14]. Notably, red blood cell (RBC) transfusion independence was achieved in 58% of patients who were transfusion dependent at baseline [14]. Importantly, responses deepened with time, with the CR rate increasing to 56% with prolonged (≥6 months) follow-up [14]. These encouraging results provided the basis for the ongoing randomized, phase 3 ENHANCE trial comparing azacitidine plus magrolimab versus placebo in patients with previously untreated HR-MDS (NCT04313881).

Among the 52 patients with treatment-naive AML treated in this phase 1b trial, 65% were *TP53* mutated, and 64% had complex karyotype [13]. Of the 34 patients evaluable for response, 65% achieved an objective response with a CR/CRi rate of 56%, of which 37% achieved MRD negativity using MFC [13]. Similar to the MDS cohort, the median time to response was 2 months. Objective responses were observed in 71% (15/21) of patients with *TP53*-mutant AML, of which 67% (14/21) of patients achieved CR/CRi [13]. Median OS for *TP53*-mutant and wild-type AML patients were 12.9 and 18.9 months, respectively [13]. The efficacy seen with this combination, particularly in *TP53*-mutant AML, led to the ongoing randomized phase 3 ENHANCE-2 trial comparing magrolimab plus azacitidine to the physician’s choice of venetoclax plus azacitidine or 7 + 3 chemotherapy in untreated *TP53*-mutant AML (NCT04778397) [74].

Although the combination of azacitidine plus venetoclax induces high CR/CRi rates (>70%), relapses are common, with less than 40% of the patients alive at 3 years [75]. Preclinical studies have shown a significant increase in phagocytosis in AML cell lines treated with the combination of magrolimab plus azacitidine and venetoclax compared to magrolimab and azacitidine plus venetoclax alone, including venetoclax-resistant and *TP53*-mutant cell lines [76]. In vivo studies, the triplet combination also improved the survival of AML PDX models, including venetoclax-resistant models. [76] Therefore, a phase Ib/II trial was conducted to evaluate the triplet regimen in both the frontline and R/R settings [77]. Thirty-eight patients were enrolled on this study with newly diagnosed (N = 17), R/R venetoclax-naïve (N = 8), and R/R venetoclax-resistant (N = 13) AML [77]. The CR/CRi rates for each cohort were 94%, 63%, and 27%, respectively, denoting the clinical activity of this triplet regimen [77]. 

Using its active Fc domain, magrolimab triggers phagocytosis through interaction with Fc gamma receptors on macrophages [71]. Due to the ubiquitous expression of CD47 on normal cells, there have been concerns about widespread toxicity with magrolimab [68]. However, since CD47 blockade only results in cell clearance in the presence of prophagocytic signals, on-target toxicity with magrolimab has been limited due to the lack of expression of prophagocytic signals on normal cells [13,14,68]. One major exception is on-target anemia. RBCs highly express CD47 as a protective mechanism against RBC clearance [78]. Senescent erythrocytes lose CD47 and gain the expression of pro-phagocytic signals leading to their physiological clearance by splenic macrophages [79,80,81]. Therefore, magrolimab induces hemolytic anemia due to the accelerated clearance of senescent erythrocytes [82,83]. This adverse event was mitigated using a priming and maintenance dose strategy for magrolimab, which eliminated older erythrocytes sparing younger RBCs, which lack prophagocytic signals. This priming dose leads to a predictable and transient mild anemia, followed by compensatory reticulocytosis shifting RBCs to a younger population that does not express significant pro-phagocytic signals, therefore unaffected by magrolimab [68,82,83]. Furthermore, the priming dose induces RBC-specific CD47 pruning through the rapid shed of cell surface CD47, rendering erythrocytes safe from subsequent doses of magrolimab [84]. In the phase 1b study, this dosing strategy resulted in mild, transient on-target anemia [13,14]. In the MDS cohort, the mean drop in hemoglobin after the first dose of magrolimab was only 0.4 g/dL [14]. Among the AML group, anemia was reported in 31% of patients, with 56% of the patients becoming transfusion independent on therapy [13]. 

### 4.2. Evorpacept (ALX148)

Evorpacept is an engineered high-affinity CD47-blocking fusion protein with an inactive modified human immunoglobulin Fc domain [85]. The inactive Fc domain is mutated to eliminate Fc gamma receptor binding, thereby preventing phagocytosis of normal blood cells and minimizing toxicity [85]. Unlike magrolimab, in vitro studies have shown that ALX148 does not cause hemagglutination of human erythrocytes [78,86]. In mouse models, the levels of RBCs, platelets, and white blood cells remained stable after administration of ALX148 but declined by 34%, 70%, and 67%, respectively, after the use of ALX377, a control protein with an identical SIRPα domain fused to an active Fc domain, confirming its more favorable preclinical safety profile [78]. 

There are two ongoing trials assessing ALX148 in combination with azacitidine in HR-MDS (ASPEN-02; NCT04417517) and in combination with azacitidine plus venetoclax in AML (ASPEN-05; NCT04755244). ASPEN-02 is a phase 1/2 multicenter study designed to assess the safety and tolerability and establish the recommended phase 2 dose (RP2D) of evorpacept when given in combination with azacitidine in the phase 1 part of the study [87]. The phase 2 portion of the trial will evaluate the efficacy of evorpacept plus azacitidine compared to azacitidine alone in patients with newly diagnosed HR-MDS [87]. Results from the phase 1 portion of the ASPEN-02 trial were recently presented [87]. Thirteen patients were enrolled, seven with newly diagnosed HR-MDS and six with R/R MDS [87]. The combination was well-tolerated with no dose-limiting toxicities observed; 60 mg/kg intravenously every 4 weeks was determined to be the RP2D of evorpacept [87]. Ten patients (five newly diagnosed and five R/R) treated at different dose levels were evaluable for response; three achieved marrow CR (including 1 with HI), three with stable disease, and two with cytogenetic response (including 1 with HI) [87]. These promising preliminary results will be further evaluated in the ongoing randomized phase 2 portion of the trial. 

## 5. TIM-3 Blockade

TIM-3 is an immune checkpoint receptor that regulates adaptive and innate immunity and is expressed on numerous immune cells, including T-cells, antigen-presenting cells, and natural killer cells [15]. TIM-3 is aberrantly expressed on the surface of LSC and blasts while sparing normal hematopoietic stem cells, with higher levels of expression associated with poor prognosis [16,88]. In MDS, TIM-3 is also expressed by blasts, with expression levels increasing at the time of disease progression and AML transformation [89,90]. Galectin-9, a TIM-3 ligand, is secreted by LSC, creating an autocrine stimulatory loop, thereby promoting LSC self-renewal [91]. In addition, galectin-9 induces apoptosis of T helper type 1 effector cells and cytotoxic TIM-3^+^ CD8^+^ T-cells, leading to T-cell exhaustion and immune evasion [92,93]. In xenograft mouse models, TIM-3 blockade led to a significant reduction in leukemic burden and eliminated LSCs making it an attractive target for the treatment of AML and HR-MDS [94].

### Sabatolimab (MBG453)

Sabatolimab, a novel anti-TIM-3 monoclonal antibody, has been found to exert its antileukemic activity through multiple mechanisms, including (1) direct targeting of TIM-3 of the surface of LSC and blasts, (2) blocking TIM-3/galaectin-9 interaction thereby preventing LSC self-renewal, and (3) promoting antibody-dependent cellular phagocytosis of LSC and blasts via binding to both TIM-3 on the surface of LSC/blasts and Fc gamma receptors on the surface of macrophages [95]. 

Sabatolimab was assessed in combination with HMAs in patients with both HR-MDS (N = 53) and newly diagnosed AML unfit for intensive chemotherapy (N = 48) [96,97]. Among the patients with MDS, the ORR was 57%, with 43% of the patients achieving CR or marrow CR [96]. Responses were durable, with a median duration of response of 16.1 months and a 1-year progression-free survival (PFS) rate of 51.9% [96]. In AML patients, the ORR was 40%, with 30% of these patients achieving CR/CRi. The median duration of response was 12.6 months with a 1-year PFS rate of 27.9% [96]. 

In subgroup analysis, responses were preserved in patients with *TP53*, *RUNX1*, and *ASXL1* mutations. In the MDS subgroup, the ORR among patients with a *TP53* mutation was 71.4% (10/14), with a median duration of response of 21.5 months, comparing favorably to historical data [96]. In patients with newly diagnosed AML with at least one ELN adverse risk mutation (*TP53*/*RUNX1*/*ASXL1*), the ORR rate was 53.8% (7/13) with a median duration of response of 12.6 months [96]. Of note, 24.5% of patients with MDS were successfully bridged to allo-SCT with favorable transplant outcomes without increased risk for GVHD [96,98]. 

Based on these encouraging results, the STIMULUS clinical trial program was launched in which several single-arm and randomized phase 2 and 3 trials are investigating multiple sabatolimab-based combination regimens in both AML and high-risk MDS and CMML (Table 1) [99]. Results from these trials will provide definitive insights into the clinical efficacy of TIM-3 blockade in the treatment of AML and HR-MDS. 

## 6. Future Directions

Given the modest single-agent activity of ICIs, combination regimens leading to dual-targeting blockade are being developed to further enhance the restoration of immune surveillance and improve outcomes for patients with AML and MDS. Inspired by the melanoma experience, double blockade of CTLA-4 and PD-1/PD-L1 is a promising combination regimen [100]. Trials assessing ipilimumab plus nivolumab in AML, MDS, and after allo-SCT are ongoing as previously described. Combined blockade of the TIM-3 and PD-1/PD-L1 pathways is another potential therapeutic combination regimen of interest. Increased coexpression of TIM-3 and PD-1 has been associated with T-cell exhaustion and AML progression [3,101,102]. In mouse models, combined blockade of the PD-1/PD-L1 and TIM-3/galectin-9 pathways resulted in a significant reduction in leukemic burden and improvement in survival compared to either agent alone, providing the rationale for their combined use in AML [101]. 

Interestingly, the adaptive immune system plays a major role in the anti-tumor activity of anti-CD47 inhibitors. Anti-CD47-mediated phagocytosis of malignant cells by antigen-presenting cells (macrophages and dendritic cells) triggers an anti-tumor cytotoxic T-cell immune response [103,104]. In preclinical studies, the anti-tumor effects of CD47-blockade were abrogated by T-cell deficiency, with substantial improvement in responses seen with the combined use of PD-L1 and CD47 antibodies [104,105]. These results support the combined use of CD47/SIRPα inhibitors and T-cell immune checkpoint inhibitors, such as PD-1/PD-L1 and TIM-3 inhibitors, to further enhance anti-tumor responses, which has been demonstrated in clinical trials conducted in patients with advanced solid tumors [106]. 

Identifying the optimal timing to introduce ICIs and the subgroup of patients with the best response to therapy is essential for the proper incorporation of ICIs in the treatment of AML and MDS. In the study conducted by Daver et al., patients with lower leukemic burden had the best response to azacitidine plus nivolumab, which was thought to be due to the higher population of bone marrow CD3^+^/CD4^+^/CD8^+^ T-cells in these patients [37]. This data suggests that T-cell-based immunotherapies are potentially most effective when used early in the course of therapy in the presence of low disease burden, such as for MRD eradication [37]. Although single-agent nivolumab failed to show clinical activity as maintenance therapy in AML, whether using it in combination with an HMA, such as oral azacitidine, would further improve OS in AML through MRD eradication is yet to be determined [41,107]. 

## 7. Conclusions

Immune checkpoint inhibition remains an area of intense clinical research in AML and MDS. CD47/SIRPα axis-targeting agents are promising additions to the current therapeutic armamentarium in myeloid malignancies. Early phase trials have shown encouraging results with the combined use of azacitidine and magrolimab in AML and HR-MDS. Randomized studies are currently ongoing to validate the efficacy of this combination and explore its potential therapeutic role in various molecular subgroups of AML and MDS, such as *TP53*-mutant disease. Sabatolimab has also shown promising clinical activity when combined with HMAs, particularly in patients with HR-MDS, with responses preserved in patients with high-risk mutations. Numerous trials are currently being conducted to further explore the role of TIM-3 blockade in myeloid malignancies. Although CTLA-4 and PD-1/PD-L1 inhibitors have not shown robust clinical activity in AML, the addition of PD-1 inhibitors to intensive chemotherapy and HMAs achieved encouraging rates of MRD negative CR, suggesting their potential role in MRD eradication. This observation is currently being investigated in randomized trials. Dual targeting of different immune checkpoints has the potential for harnessing the immune system and improving anti-leukemic responses. Clinical trials exploring the synergy between various combinations of ICIs and HMAs or cytotoxic chemotherapy are underway.

## Figures and Tables

**Table 1:** Overview of Ongoing Trials of Immune Checkpoint Inhibitors in Acute Myeloid Leukemia and Myelodysplastic Syndromes.

Target	Agent	Regimen	Study Population	Phase	NCT Identifier
PD-1	Nivolumab	Nivolumab + Ipilimumab	R/R AML and MDS following Allo-SCT	1b	NCT03600155
		AZA + Nivolumab ± Ipilimumab	ND and R/R AML	2	NCT02397720
		Nivolumab ± Ipilimumab ± AZA	ND and R/R MDS	2	NCT02530463
	Pembrolizumab	IC ± Pembrolizumab (BLAST MRD AML-1)	ND AML	2	NCT04214249
		AZA + VEN ± Pembrolizumab (BLAST MRD AML-2)	ND AML	2	NCT04284787
CD47-SIPRa	Magrolimab (Hu5F9-G4)	Magrolimab ± AZA	ND and R/R AML and HR-MDS	1b	NCT03248479
		AZA + VEN + Magrolimab	ND and R/R AML	1b/2	NCT04435691
		Magrolimab + AZA/VEN (Cohort 1); MEC (Cohort 2); or CC-486 (Cohort 3)	ND, R/R, and MRD + AML	2	NCT04778410
		AZA + Magrolimab vs. Placebo (ENHANCE)	ND HR-MDS	3	NCT04313881
		AZA + Magrolimab vs. AZA/VEN or IC (Physician Choice) (ENHANCE-2)	ND *TP53*-mutant AML	3	NCT04778397
		AZA + VEN + Magrolimab versus Placebo (ENHANCE-3)	ND AML	3	NCT05079230
	Evorpacept (ALX148)	AZA + Evorpacept (ASPEN-2)	ND and R/R HR-MDS	1/2	NCT04417517
		AZA + VEN + Evorpacept (ASPEN-5)	ND and R/R AML	1/2	NCT04755244
	TTI-622	TTI-622 + AZA ± VEN	ND AML	1	NCT03530683
	TTI-621	TTI-621 monotherapy	AML and MDS	1	NCT02663518
TIM-3	Sabatolimab (MBG453)	HMA + Sabatolimab vs. Placebo (STIMULUS-MDS1)	ND HR-MDS	2	NCT03946670
		AZA + Sabatolimab vs. Placebo (STIMULUS-MDS2)	ND HR-MDS and CMML-2	3	NCT04266301
		HMA + Sabatolimab (STIMULUS MDS-US)	ND HR-MDS	2	NCT04878432
		AZA + VEN + Sabatolimab (STIMULUS-MDS3)	ND HR-MDS	2	NCT04812548
		Sabatolimab monotherapy	LR-MDS	2	NCT04823624
		Sabatolimab ± NIS793 or Canakinumab	LR-MDS	1b	NCT04810611
		AZA + VEN + Sabatolimab (STIMULUS-AML1)	ND AML	2	NCT04150029
		Sabatolimab ± AZA	AML with MRD + CR after Allo-SCT	1b/2	NCT04623216

Abbreviations: Allo-SCT: allogeneic stem cell transplantation; AML: acute myeloid leukemia; AZA: azacitidine; CMML: chronic myelomonocytic leukemia; HMA: hypomethylating agent; HR-MDS: higher-risk myelodysplastic syndromes; IC: intensive chemotherapy; LR: lower-risk; MEC: mitoxantrone/etoposide/cyclophosphamide; MRD: measurable residual disease; ND: newly diagnosed; PD-1: programmed cell death-1; R/R: relapsed/refractory; VEN: venetoclax.

**Table 2:** CD47-SIRPα Targeting Agents under Investigation in Myeloid Neoplasms.

Agent	Mechanism of Action	Fc Domain	RBC Sparing	Trial Phase	Study Population
Magrolimab (Hu5F9-G4)	Humanized anti-CD47 mAb	Active IgG4	No	3	AML/MDS
Evorpacept (ALX148)	High affinity CD47-binding SIRPa-Fc fusion protein (decoy receptor)	Inactive IgG1	No	1/2	AML/MDS
TTI-621	Anti-CD47 SIRPα-Fc fusion protein (decoy receptor)	Active IgG1	Yes	1	Advanced Hematologic Malignancies
TTI-622	Anti-CD47 SIRPα-Fc fusion protein (decoy receptor)	Active IgG4	Yes	1	Advanced Hematologic Malignancies
Lemzoparlimab (TJ011133)	Highly differentiated fully human anti-CD47 mAb	Active IgG4	Yes	1	AML/MDS (NCT04912063)
SL-172154	SIRPα-Fc-CD40L fusion protein	Inactive IgG4	Yes	1	AML/MDS (NCT05275439)
CC-95251	Fully human Anti-SIRPα mAb	Active IgG1	Yes	1	AML/MDS (NCT05168202)

Abbreviations: Fc: fragment crystallizable; AML: acute myeloid leukemia; MDS: myelodysplastic syndromes; mAb: monoclonal antibody; SIRPα: signal regulatory protein alpha.

## References

[1] Horowitz M.M., Gale R.P., Sondel P.M., Goldman J.M., Kersey J., Kolb H.J., Rimm A.A., Ringden O., Rozman C., Speck B. (1990). Graft-versus-leukemia reactions after bone marrow transplantation. Blood.

[2] Vasu S., Kohlschmidt J., Mrózek K., Eisfeld A.-K., Nicolet D., Sterling L.J., Becker H., Metzeler K., Papaioannou D., Powell B.L. (2018). Ten-year outcome of patients with acute myeloid leukemia not treated with allogeneic transplantation in first complete remission. Blood Adv..

[3] Williams P., Basu S., Garcia-Manero G., Hourigan C.S., Oetjen K.A., Cortes J.E., Ravandi F., Jabbour E.J., Al-Hamal Z., Konopleva M. (2019). The distribution of T-cell subsets and the expression of immune checkpoint receptors and ligands in patients with newly diagnosed and relapsed acute myeloid leukemia. Cancer.

[4] Wei S.C., Duffy C.R., Allison J.P. (2018). Fundamental Mechanisms of Immune Checkpoint Blockade Therapy. Cancer Discov..

[5] Vaddepally R.K., Kharel P., Pandey R., Garje R., Chandra A.B. (2020). Review of Indications of FDA-Approved Immune Checkpoint Inhibitors per NCCN Guidelines with the Level of Evidence. Cancers.

[6] Giannopoulos K. (2019). Targeting Immune Signaling Checkpoints in Acute Myeloid Leukemia. J. Clin. Med..

[7] El Hussein S., Daver N., Liu J.-L., Kornblau S., Fang H., Konoplev S., Kantarjian H., Khoury J.D. (2022). Microsatellite Instability Assessment by Immunohistochemistry in Acute Myeloid Leukemia: A Reappraisal and Review of the Literature. Clin. Lymphoma Myeloma Leuk..

[8] Le D.T., Uram J.N., Wang H., Bartlett B.R., Kemberling H., Eyring A.D., Skora A.D., Luber B.S., Azad N.S., Laheru D. (2015). PD-1 Blockade in Tumors with Mismatch-Repair Deficiency. N. Engl. J. Med..

[9] Lyu G.-Y., Yeh Y.-H., Yeh Y.-C., Wang Y.-C. (2018). Mutation load estimation model as a predictor of the response to cancer immunotherapy. NPJ Genom. Med..

[10] Martincorena I., Campbell P.J. (2015). Somatic mutation in cancer and normal cells. Science.

[11] Chao M.P., Weissman I.L., Majeti R. (2012). The CD47–SIRPα pathway in cancer immune evasion and potential therapeutic implications. Curr. Opin. Immunol..

[12] Majeti R., Chao M.P., Alizadeh A.A., Pang W.W., Jaiswal S., Gibbs K.D., Van Rooijen N., Weissman I.L. (2009). CD47 Is an Adverse Prognostic Factor and Therapeutic Antibody Target on Human Acute Myeloid Leukemia Stem Cells. Cell.

[13] Sallman D.A., Asch A.S., Kambhampati S., Al Malki M.M., Zeidner J.F., Donnellan W., Lee D.J., Vyas P., Jeyakumar D., Mannis G.N. (2020). The First-in-Class Anti-CD47 Antibody Magrolimab Combined with Azacitidine Is Well-Tolerated and Effective in AML Patients: Phase 1b Results. Blood.

[14] Sallman D.A., Asch A.S., Kambhampati S., Al Malki M.M., Zeidner J.F., Donnellan W., Vyas P., Pollyea D., Bradley T., Jeyakumar D. (2020). The First-in-Class Anti-CD47 Antibody Magrolimab Combined with Azacitadine is Well-Tolerated and Effective in MDS Patients: PHASE 1B Results.

[15] Das M., Zhu C., Kuchroo V.K. (2017). Tim-3 and its role in regulating anti-tumor immunity. Immunol. Rev..

[16] Jan M., Chao M.P., Cha A.C., Alizadeh A.A., Gentles A.J., Weissman I.L., Majeti R. (2011). Prospective separation of normal and leukemic stem cells based on differential expression of TIM3, a human acute myeloid leukemia stem cell marker. Proc. Natl. Acad. Sci. USA.

[17] Alatrash G., Daver N., Mittendorf E.A. (2016). Targeting Immune Checkpoints in Hematologic Malignancies. Pharmacol. Rev..

[18] Costello R.T., Mallet F., Sainty D., Maraninchi D., Gastaut J.A., Olive D. (1998). Regulation of CD80/B7-1 and CD86/B7-2 molecule expression in human primary acute myeloid leukemia and their role in allogenic immune recognition. Eur. J. Immunol..

[19] Graf M., Reif S., Hecht K., Pelka-Fleischer R., Kroell T., Pfister K., Schmetzer H. (2005). High expression of costimulatory molecules correlates with low relapse-free survival probability in acute myeloid leukemia (AML). Ann. Hematol..

[20] Davids M.S., Kim H.T., Bachireddy P., Costello C., Liguori R., Savell A., Lukez A.P., Avigan D., Chen Y.-B., McSweeney P. (2016). Ipilimumab for Patients with Relapse after Allogeneic Transplantation. N. Engl. J. Med..

[21] Zeidan A.M., Knaus H.A., Robinson T.M., Towlerton A.M.H., Warren E.H., Zeidner J.F., Blackford A.L., Duffield A.S., Rizzieri D., Frattini M.G. (2018). A Multi-center Phase I Trial of Ipilimumab in Patients with Myelodysplastic Syndromes following Hypomethylating Agent Failure. Clin. Cancer Res..

[22] Toffalori C., Cavattoni I., Deola S., Mastaglio S., Giglio F., Mazzi B., Assanelli A., Peccatori J., Bordignon C., Bonini C. (2012). Genomic loss of patient-specific HLA in acute myeloid leukemia relapse after well-matched unrelated donor HSCT. Blood.

[23] Vago L., Perna S.K., Zanussi M., Mazzi B., Barlassina C., Stanghellini M.T.L., Perrelli N.F., Cosentino C., Torri F., Angius A. (2009). Loss of Mismatched HLA in Leukemia after Stem-Cell Transplantation. N. Engl. J. Med..

[24] Urban J.L., Schreiber H. (1992). Tumor Antigens. Annu. Rev. Immunol..

[25] Blank C., Brown I., Peterson A.C., Spiotto M., Iwai Y., Honjo T., Gajewski T.F. (2004). PD-L1/B7H-1 Inhibits the Effector Phase of Tumor Rejection by T Cell Receptor (TCR) Transgenic CD8+ T Cells. Cancer Res..

[26] Chemnitz J.M., Parry R.V., Nichols K.E., June C., Riley J.L. (2004). SHP-1 and SHP-2 Associate with Immunoreceptor Tyrosine-Based Switch Motif of Programmed Death 1 upon Primary Human T Cell Stimulation, but Only Receptor Ligation Prevents T Cell Activation. J. Immunol..

[27] Dong H., Strome S.E., Salomao D.R., Tamura H., Hirano F., Flies D.B., Roche P.C., Lu J., Zhu G., Tamada K. (2002). Tumor-associated B7-H1 promotes T-cell apoptosis: A potential mechanism of immune evasion. Nat. Med..

[28] Zhang L., Gajewski T.F., Kline J. (2009). PD-1/PD-L1 interactions inhibit antitumor immune responses in a murine acute myeloid leukemia model. Blood.

[29] Zhou Q., Munger M., Highfill S.L., Tolar J., Weigel B.J., Riddle M., Sharpe A.H., Vallera D.A., Azuma M., Levine B.L. (2010). Program death-1 signaling and regulatory T cells collaborate to resist the function of adoptively transferred cytotoxic T lymphocytes in advanced acute myeloid leukemia. Blood.

[30] Chen X., Liu S., Wang L., Zhang W.-G., Ji Y., Ma X. (2008). Clinical significance of B7-H1（PD-L1）expression in human acute leukemia. Cancer Biol. Ther..

[31] Sallman D.A., McLemore A.F., Aldrich A.L., Komrokji R.S., McGraw K.L., Dhawan A., Geyer S., Hou H.-A., Eksioglu E.A., Sullivan A. (2020). *TP53* mutations in myelodysplastic syndromes and secondary AML confer an immunosuppressive phenotype. Blood.

[32] Berger R., Rotem-Yehudar R., Slama G., Landes S., Kneller A., Leiba M., Koren-Michowitz M., Shimoni A., Nagler A. (2008). Phase I Safety and Pharmacokinetic Study of CT-011, a Humanized Antibody Interacting with PD-1, in Patients with Advanced Hematologic Malignancies. Clin. Cancer Res..

[33] Yang H., Bueso-Ramos C., Dinardo C., Estecio M.R., Davanlou M., Geng Q.-R., Fang Z., Nguyen M., Pierce S., Wei Y. (2014). Expression of PD-L1, PD-L2, PD-1 and CTLA4 in myelodysplastic syndromes is enhanced by treatment with hypomethylating agents. Leukemia.

[34] Wang L.-X., Mei Z.-Y., Zhou J.-H., Yao Y.-S., Li Y.-H., Xu Y.-H., Li J.-X., Gao X.-N., Zhou M.-H., Jiang M.-M. (2013). Low Dose Decitabine Treatment Induces CD80 Expression in Cancer Cells and Stimulates Tumor Specific Cytotoxic T Lymphocyte Responses. PLoS ONE.

[35] Weber J., Salgaller M., Samid D., Johnson B., Herlyn M., Lassam N., Treisman J., Rosenberg S.A. (1994). Expression of the MAGE-1 tumor antigen is up-regulated by the demethylating agent 5-aza-2'-deoxycytidine. Cancer Res..

[36] Vereecque R., Saudemont A., Quesnel B. (2004). Cytosine arabinoside induces costimulatory molecule expression in acute myeloid leukemia cells. Leukemia.

[37] Daver N., Garcia-Manero G., Basu S., Boddu P.C., Alfayez M., Cortes J.E., Konopleva M., Ravandi-Kashani F., Jabbour E., Kadia T. (2019). Efficacy, Safety, and Biomarkers of Response to Azacitidine and Nivolumab in Relapsed/Refractory Acute Myeloid Leukemia: A Nonrandomized, Open-Label, Phase II Study. Cancer Discov..

[38] Postow M.A., Sidlow R., Hellmann M.D. (2018). Immune-Related Adverse Events Associated with Immune Checkpoint Blockade. N. Engl. J. Med..

[39] Daver N.G., Garcia-Manero G., Konopleva M.Y., Alfayez M., Pemmaraju N., Kadia T.M., Dinardo C.D., Cortes J.E., Ravandi F., Abbas H. (2019). Azacitidine (AZA) with Nivolumab (Nivo), and AZA with Nivo + Ipilimumab (Ipi) in Relapsed/Refractory Acute Myeloid Leukemia: A Non-Randomized, Prospective, Phase 2 Study. Blood.

[40] Ravandi F., Assi R., Daver N., Benton C.B., Kadia T., Thompson P.A., Borthakur G., Alvarado Y., Jabbour E.J., Konopleva M. (2019). Idarubicin, cytarabine, and nivolumab in patients with newly diagnosed acute myeloid leukaemia or high-risk myelodysplastic syndrome: A single-arm, phase 2 study. Lancet Haematol..

[41] Reville P.K., Kantarjian H.M., Ravandi F., Jabbour E., DiNardo C.D., Daver N., Pemmaraju N., Ohanian M., Alvarado Y., Xiao L. (2021). Nivolumab maintenance in high-risk acute myeloid leukemia patients: A single-arm, open-label, phase II study. Blood Cancer J..

[42] Gojo I., Stuart R.K., Webster J., Blackford A., Varela J.C., Morrow J., DeZern A.E., Foster M.C., Levis M.J., Coombs C.C. (2019). Multi-Center Phase 2 Study of Pembroluzimab (Pembro) and Azacitidine (AZA) in Patients with Relapsed/Refractory Acute Myeloid Leukemia (AML) and in Newly Diagnosed (≥65 Years) AML Patients. Blood.

[43] Goswami M., Gui G., Dillon L.W., Lindblad K.E., Thompson J., Valdez J., Kim D.-Y., Ghannam J.Y., Oetjen K.A., Destefano C.B. (2022). Pembrolizumab and decitabine for refractory or relapsed acute myeloid leukemia. J. Immunother. Cancer.

[44] Zeidner J.F., Vincent B.G., Ivanova A., Moore D.T., McKinnon K.P., Wilkinson A.D., Mukhopadhyay R., Mazziotta F., Knaus H.A., Foster M.C. (2021). Phase II Trial of Pembrolizumab after High-Dose Cytarabine in Relapsed/Refractory Acute Myeloid Leukemia. Blood Cancer Discov..

[45] Tschernia N.P., Kumar V., Moore D.T., Vincent B.G., Coombs C.C., Van Deventer H., Foster M.C., DeZern A.E., Luznik L., Riches M.L. (2021). Safety and Efficacy of Pembrolizumab Prior to Allogeneic Stem Cell Transplantation for Acute Myelogenous Leukemia. Transplant. Cell. Ther..

[46] Saberian C., Abdel-Wahab N., Abudayyeh A., Rafei H., Joseph J., Rondon G., Whited L., Gruschkus S., Fa'Ak F., Daher M. (2021). Post-transplantation cyclophosphamide reduces the incidence of acute graft-versus-host disease in patients with acute myeloid leukemia/myelodysplastic syndromes who receive immune checkpoint inhibitors after allogeneic hematopoietic stem cell transplantation. J. Immunother. Cancer.

[47] Oran B., Garcia-Manero G., Saliba R.M., Alfayez M., Do G.A., Ciurea S.O., Jabbour E.J., Mehta R.S., Popat U.R., Ravandi F. (2020). Posttransplantation cyclophosphamide improves transplantation outcomes in patients with AML/MDS who are treated with checkpoint inhibitors. Cancer.

[48] Schoch L.K., Cooke K.R., Wagner-Johnston N.D., Gojo I., Swinnen L.J., Imus P., Fuchs E.J., Levis M., Ambinder R.F., Jones R.J. (2018). Immune checkpoint inhibitors as a bridge to allogeneic transplantation with posttransplant cyclophosphamide. Blood Adv..

[49] Garcia-Manero G., Ribrag V., Zhang Y., Farooqui M., Marinello P., Smith B.D. (2022). Pembrolizumab for myelodysplastic syndromes after failure of hypomethylating agents in the phase 1b KEYNOTE-013 study. Leuk. Lymphoma.

[50] Gil-Perez A., Montalban-Bravo G. (2019). Management of myelodysplastic syndromes after failure of response to hypomethylating agents. Ther. Adv. Hematol..

[51] Jabbour E., Garcia-Manero G., Batty N., Shan J., O'Brien S., Cortes J., Ravandi F., Issa J.-P., Kantarjian H. (2010). Outcome of patients with myelodysplastic syndrome after failure of decitabine therapy. Cancer.

[52] Chien K.S., Kim K., Nogueras-Gonzalez G.M., Borthakur G., Naqvi K., Daver N.G., Montalban-Bravo G., Cortes J.E., DiNardo C.D., Jabbour E. (2021). Phase II study of azacitidine with pembrolizumab in patients with intermediate-1 or higher-risk myelodysplastic syndrome. Br. J. Haematol..

[53] Brahmer J.R., Lacchetti C., Schneider B.J., Atkins M.B., Brassil K.J., Caterino J.M., Chau I., Ernstoff M.S., Gardner J.M., Ginex P. (2018). Management of immune-related adverse events in patients treated with immune checkpoint inhibitor therapy: American Society of Clinical Oncology Clinical Practice Guideline. J. Clin. Oncol..

[54] Shallis R.M., Bewersdorf J.P., Swoboda D.M., Wei W., Gowda L., Prebet T., Halene S., Pillai M.M., Parker T., Neparidze N. (2021). Challenges in the Evaluation and Management of Toxicities Arising From Immune Checkpoint Inhibitor Therapy for Patients With Myeloid Malignancies. Clin. Lymphoma Myeloma Leuk..

[55] Zheng H., Mineishi S., Claxton D., Zhu J., Zhao C., Jia B., Ehmann W.C., Rybka W.B., Naik S., Songdej N. (2021). A phase I clinical trial of avelumab in combination with decitabine as first line treatment of unfit patients with acute myeloid leukemia. Am. J. Hematol..

[56] Kantarjian H.M., Thomas X.G., Dmoszynska A., Wierzbowska A., Mazur G., Mayer J., Gau J.-P., Chou W.-C., Buckstein R., Cermak J. (2012). Multicenter, Randomized, Open-Label, Phase III Trial of Decitabine Versus Patient Choice, With Physician Advice, of Either Supportive Care or Low-Dose Cytarabine for the Treatment of Older Patients With Newly Diagnosed Acute Myeloid Leukemia. J. Clin. Oncol..

[57] Zeidan A.M., Boss I.W., Beach C.L., Copeland W.B., Thompson E.G., Fox B.A., Hasle V.E., Hellmann A., Taussig D.C., Tormo M. (2022). A randomized phase 2 trial of azacitidine with or without durvalumab as first-line therapy for older patients with AML. Blood Adv..

[58] Saxena K., Herbrich S.M., Pemmaraju N., Kadia T.M., DiNardo C.D., Borthakur G., Pierce S.A., Jabbour E., Wang S.A., Bueso-Ramos C. (2021). A phase 1b/2 study of azacitidine with PD-L1 antibody avelumab in relapsed/refractory acute myeloid leukemia. Cancer.

[59] Stahl M., Deveaux M., Montesinos P., Itzykson R., Ritchie E.K., Sekeres M., Barnard J.D., Podoltsev N.A., Brunner A.M., Komrokji R.S. (2018). Hypomethylating agents in relapsed and refractory AML: Outcomes and their predictors in a large international patient cohort. Blood Adv..

[60] Zeidan A.M., Boss I.W., Beach C., Copeland W.B., Thompson E.G., Fox B.A., Hasle V.E., Ogasawara K., Cavenagh J., Silverman L.R. (2022). A Randomized Phase 2 Trial of Azacitidine ± Durvalumab as First-line Therapy for Higher-Risk Myelodysplastic Syndromes. Blood Adv..

[61] Gerds A.T., Scott B.L., Greenberg P.L., Lin T.L., Pollyea D.A., Verma A.K., Dail M., Feng Y., Green C., Ma C. (2022). Atezolizumab alone or in combination did not demonstrate a favorable risk-benefit profile in myelodysplastic syndrome. Blood Adv..

[62] Tsai R.K., Discher D.E. (2008). Inhibition of “self” engulfment through deactivation of myosin-II at the phagocytic synapse between human cells. J. Cell Biol..

[63] Chao M.P., Jaiswal S., Weissman-Tsukamoto R., Alizadeh A.A., Gentles A.J., Volkmer J., Weiskopf K., Willingham S.B., Raveh T., Park C.Y. (2010). Calreticulin Is the Dominant Pro-Phagocytic Signal on Multiple Human Cancers and Is Counterbalanced by CD47. Sci. Transl. Med..

[64] Pang W.W., Pluvinage J.V., Price E.A., Sridhar K., Arber D.A., Greenberg P.L., Schrier S.L., Park C.Y., Weissman I.L. (2013). Hematopoietic stem cell and progenitor cell mechanisms in myelodysplastic syndromes. Proc. Natl. Acad. Sci. USA.

[65] Jaiswal S., Jamieson C.H., Pang W.W., Park C.Y., Chao M.P., Majeti R., Traver D., van Rooijen N., Weissman I.L. (2009). CD47 Is Upregulated on Circulating Hematopoietic Stem Cells and Leukemia Cells to Avoid Phagocytosis. Cell.

[66] Jiang H., Fu R., Wang H., Li L., Liu H., Shao Z. (2013). CD47 is expressed abnormally on hematopoietic cells in myelodysplastic syndrome. Leuk. Res..

[67] Ostendorf B.N., Flenner E., Flörcken A., Westermann J. (2018). Phenotypic characterization of aberrant stem and progenitor cell populations in myelodysplastic syndromes. PLoS ONE.

[68] Liu J., Wang L., Zhao F., Tseng S., Narayanan C., Shura L., Willingham S., Howard M., Prohaska S., Volkmer J. (2015). Pre-Clinical Development of a Humanized Anti-CD47 Antibody with Anti-Cancer Therapeutic Potential. PLoS ONE.

[69] Jiang Z., Sun H., Yu J., Tian W., Song Y. (2021). Targeting CD47 for cancer immunotherapy. J. Hematol. Oncol..

[70] Vyas P., Knapper S., Kelly R., Salim R., Lubowiecki M., Royston D., Johnson H., Roberts C., Chen J., Agoram B. (2018). Initial Phase 1 Results of the First-in-Class Anti-CD47 Antibody Hu5F9-G4 in Relapsed/Refractory Acute Myeloid Leukemia Patients.

[71] Chao M.P., Takimoto C.H., Feng D.D., McKenna K., Gip P., Liu J., Volkmer J.-P., Weissman I.L., Majeti R. (2020). Therapeutic Targeting of the Macrophage Immune Checkpoint CD47 in Myeloid Malignancies. Front. Oncol..

[72] Feng D., Gip P., McKenna B.K.M., Zhao F., Mata O., Choi T., Duan M.J., Sompalli M.K., Majeti R., Weissman I.L. (2018). Combination Treatment with 5F9 and Azacitidine Enhances Phagocytic Elimination of Acute Myeloid Leukemia. Blood.

[73] Boasman K., Bridle C., Simmonds M.J., Rinaldi C.R. (2017). Role of Pro-phagocytic Calreticulin and Anti-phagocytic CD47 in MDS and MPN Models Treated with Azacytidine or Ruxolitinib.

[74] Daver N., Vyas P., Chao M., Xing G., Renard C., Ramsingh G., Sallman D.A., Wei A.H. (2021). A Phase 3, Randomized, Open-Label Study Evaluating the Safety and Efficacy of Magrolimab in Combination with Azacitidine in Previously Untreated Patients with *TP53*-Mutant Acute Myeloid Leukemia. Blood.

[75] Pollyea D.A., Pratz K., Letai A., Jonas B.A., Wei A.H., Pullarkat V., Konopleva M., Thirman M.J., Arellano M., Becker P.S. (2021). Venetoclax with azacitidine or decitabine in patients with newly diagnosed acute myeloid leukemia: Long term follow-up from a phase 1b study. Am. J. Hematol..

[76] Jia Y., Zhang Q., Weng C., Ramage C.L., Nishida Y., Chao M., Maute R.L., Herbrich S., Zhang W., Andreeff M. (2021). Combined Blockade of CD47-Sirpa Interaction By 5F9 (Magrolimab) and Azacitidine/Venetoclax Therapy Facilitates Macrophage-Mediated Anti-Leukemia Efficacy in AML Pre-Clinical Models. Blood.

[77] Daver N., Konopleva M., Maiti A., Kadia T.M., DiNardo C.D., Loghavi S., Pemmaraju N., Jabbour E.J., Montalban-Bravo G., Tang G. (2021). Phase I/II Study of Azacitidine (AZA) with Venetoclax (VEN) and Magrolimab (Magro) in Patients (pts) with Newly Diagnosed Older/Unfit or High-Risk Acute Myeloid Leukemia (AML) and Relapsed/Refractory (R/R) AML. Blood.

[78] Kauder S.E., Kuo T.C., Harrabi O., Chen A., Sangalang E., Doyle L., Rocha S.S., Bollini S., Han B., Sim J. (2018). ALX148 blocks CD47 and enhances innate and adaptive antitumor immunity with a favorable safety profile. PLoS ONE.

[79] Oldenborg P.-A., Zheleznyak A., Fang Y.-F., Lagenaur C.F., Gresham H.D., Lindberg F.P. (2000). Role of CD47 as a Marker of Self on Red Blood Cells. Science.

[80] Olsson M., Bruhns P., Frazier W.A., Ravetch J.V., Oldenborg P.-A. (2005). Platelet homeostasis is regulated by platelet expression of CD47 under normal conditions and in passive immune thrombocytopenia. Blood.

[81] Khandelwal S., van Rooijen N., Saxena R.K. (2007). Reduced expression of CD47 during murine red blood cell (RBC) senescence and its role in RBC clearance from the circulation. Transfusion.

[82] Advani R., Flinn I., Popplewell L., Forero A., Bartlett N.L., Ghosh N., Kline J., Roschewski M., LaCasce A., Collins G.P. (2018). CD47 Blockade by Hu5F9-G4 and Rituximab in Non-Hodgkin’s Lymphoma. N. Engl. J. Med..

[83] Sikic B.I., Lakhani N., Patnaik A., Shah S.A., Chandana S.R., Rasco D., Colevas A.D., O’Rourke T., Narayanan S., Papadopoulos K. (2019). First-in-Human, First-in-Class Phase I Trial of the Anti-CD47 Antibody Hu5F9-G4 in Patients With Advanced Cancers. J. Clin. Oncol..

[84] Chen J.Y., McKenna B.K.M., Choi T., Duan M.J., Brown M.L., Stewart J.J., Sompalli M.K., Vyas P., Schrier S., Majeti R. (2018). RBC-Specific CD47 Pruning Confers Protection and Underlies the Transient Anemia in Patients Treated with Anti-CD47 Antibody 5F9. Blood.

[85] Kim T.M., Lakhani N., Gainor J., Kamdar M., Fanning P., Squifflet P., Jin F., Wan H., Pons J., Randolph S. (2020). ALX148, A CD47 Blocker, in Combination with Rituximab in Patients with Relapsed/Refractory (R/R) Non-Hodgkin Lymphoma (NHL).

[86] Petrova P.S., Viller N.N., Wong M., Pang X., Lin G.H.Y., Dodge K., Chai V., Chen H., Lee V., House V. (2017). TTI-621 (SIRPαFc): A CD47-Blocking Innate Immune Checkpoint Inhibitor with Broad Antitumor Activity and Minimal Erythrocyte Binding. Clin. Cancer Res..

[87] Garcia-Manero G., Erba H.P., Sanikommu S.R., Altman J.K., Sayar H., Scott B.L., Fong A.P., Guan S., Jin F., Forgie A.J. (2021). Evorpacept (ALX148), a CD47-Blocking Myeloid Checkpoint Inhibitor, in Combination with Azacitidine: A Phase 1/2 Study in Patients with Myelodysplastic Syndrome (ASPEN-02). Blood.

[88] Li C., Chen X., Yu X., Zhu Y., Ma C., Xia R., Ma J., Gu C., Ye L., Wu D. (2014). Tim-3 is highly expressed in T cells in acute myeloid leukemia and associated with clinicopathological prognostic stratification. Int. J. Clin. Exp. Pathol..

[89] Asayama T., Tamura H., Ishibashi M., Kuribayashi-Hamada Y., Onodera-Kondo A., Okuyama N., Yamada A., Shimizu M., Moriya K., Takahashi H. (2017). Functional expression of Tim-3 on blasts and clinical impact of its ligand galectin-9 in myelodysplastic syndromes. Oncotarget.

[90] Kikushige Y., Miyamoto T., Yuda J., Jabbarzadeh-Tabrizi S., Shima T., Takayanagi S.-I., Niiro H., Yurino A., Miyawaki K., Takenaka K. (2015). A TIM-3/Gal-9 Autocrine Stimulatory Loop Drives Self-Renewal of Human Myeloid Leukemia Stem Cells and Leukemic Progression. Cell Stem Cell.

[91] Silva I.G., Yasinska I.M., Sakhnevych S.S., Fiedler W., Wellbrock J., Bardelli M., Varani L., Hussain R., Siligardi G., Ceccone G. (2017). The Tim-3-galectin-9 Secretory Pathway is Involved in the Immune Escape of Human Acute Myeloid Leukemia Cells. EBioMedicine.

[92] Kang C.-W., Dutta A., Yung-Chang L., Mahalingam J., Lin Y.-C., Chiang J.-M., Hsu C.-Y., Huang C.-T., Su W.-T., Chun-Yen L. (2015). Apoptosis of tumor infiltrating effector TIM-3+CD8+ T cells in colon cancer. Sci. Rep..

[93] Zhu C., Anderson A.C., Schubart A., Xiong H., Imitola J., Khoury S., Zheng X.X., Strom T.B., Kuchroo V.K. (2005). The Tim-3 ligand galectin-9 negatively regulates T helper type 1 immunity. Nat. Immunol..

[94] Kikushige Y., Shima T., Takayanagi S.-I., Urata S., Miyamoto T., Iwasaki H., Takenaka K., Teshima T., Tanaka T., Inagaki Y. (2010). TIM-3 Is a Promising Target to Selectively Kill Acute Myeloid Leukemia Stem Cells. Cell Stem Cell.

[95] Acharya N., Sabatos-Peyton C., Anderson A.C. (2020). Tim-3 finds its place in the cancer immunotherapy landscape. J. Immunother. Cancer.

[96] Brunner A.M., Esteve J., Porkka K., Knapper S., Traer E., Scholl S., Garcia-Manero G., Vey N., Wermke M., Janssen J. (2021). Efficacy and Safety of Sabatolimab (MBG453) in Combination with Hypomethylating Agents (HMAs) in Patients (Pts) with Very High/High-Risk Myelodysplastic Syndrome (vHR/HR-MDS) and Acute Myeloid Leukemia (AML): Final Analysis from a Phase Ib Study. Blood.

[97] Wei A., Esteve J., Porkka K., Knapper S., Traer E., Scholl S., Garcia-Manero G., Vey N., Wermke M., Janssen J., Wei A. (2021). Sabatolimab plus Hypomethylating Agents (HMAS) in Patients with High-/Very High-Risk Myelodysplastic Syndrome (HR/VHR-MDS) and Acute Myeloid Leukemia (AML): Subgroup Analysis of a Phase 1 Study.

[98] Brunner A.M., Traer E., Vey N., Scholl S., Tovar N., Porkka K., Narayan R., Garcia-Manero G., Knapper S., Wermke M. (2021). Allogeneic Hematopoietic Cell Transplantation Outcomes of Patients with R/R AML or Higher-Risk MDS Treated with the TIM-3 Inhibitor MBG453 (Sabatolimab) and Hypomethylating Agents. Blood.

[99] Zeidan A.M., Al-Kali A., Borate U., Cluzeau T., DeZern A.E., Esteve J., Giagounidis A., Kobata K., Lyons R., Platzbecker U. (2021). Sabatolimab (MBG453) Combination Treatment Regimens for Patients (Pts) with Higher-Risk Myelodysplastic Syndromes (HR-MDS): The MDS Studies in the Stimulus Immuno-Myeloid Clinical Trial Program. Blood.

[100] Larkin J., Chiarion-Sileni V., Gonzalez R., Grob J.-J., Cowey C.L., Lao C.D., Schadendorf D., Dummer R., Smylie M., Rutkowski P. (2015). Combined Nivolumab and Ipilimumab or Monotherapy in Untreated Melanoma. N. Engl. J. Med..

[101] Zhou Q., Munger M., Veenstra R.G., Weigel B.J., Hirashima M., Munn D., Murphy W.J., Azuma M., Anderson A.C., Kuchroo V.K. (2011). Coexpression of Tim-3 and PD-1 identifies a CD8+ T-cell exhaustion phenotype in mice with disseminated acute myelogenous leukemia. Blood.

[102] Kong Y., Zhang J., Claxton D.F., Ehmann W.C., Rybka W.B., Zhu L., Zeng H., Schell T.D., Zheng H. (2015). PD-1(hi)TIM-3(+) T cells associate with and predict leukemia relapse in AML patients post allogeneic stem cell transplantation. Blood Cancer J..

[103] Tseng D., Volkmer J.-P., Willingham S.B., Contreras-Trujillo H., Fathman J.W., Fernhoff N.B., Seita J., Inlay M.A., Weiskopf K., Miyanishi M. (2013). Anti-CD47 antibody–mediated phagocytosis of cancer by macrophages primes an effective antitumor T-cell response. Proc. Natl. Acad. Sci. USA.

[104] Liu X., Pu Y., Cron K.R., Deng L., Kline J., Frazier W.A., Xu H., Peng H., Fu Y.-X., Xu M.M. (2015). CD47 blockade triggers T cell–mediated destruction of immunogenic tumors. Nat. Med..

[105] Sockolosky J.T., Dougan M., Ingram J.R., Ho C.C.M., Kauke M.J., Almo S.C., Ploegh H.L., Garcia K.C. (2016). Durable antitumor responses to CD47 blockade require adaptive immune stimulation. Proc. Natl. Acad. Sci. USA.

[106] Lakhani N.J., Chow L.Q.M., Gainor J.F., LoRusso P., Lee K.-W., Chung H.C., Lee J., Bang Y.-J., Hodi F.S., Kim W.S. (2021). Evorpacept alone and in combination with pembrolizumab or trastuzumab in patients with advanced solid tumours (ASPEN-01): A first-in-human, open-label, multicentre, phase 1 dose-escalation and dose-expansion study. Lancet Oncol..

[107] Wei A.H., Döhner H., Pocock C., Montesinos P., Afanasyev B., Dombret H., Ravandi F., Sayar H., Jang J.-H., Porkka K. (2020). Oral Azacitidine Maintenance Therapy for Acute Myeloid Leukemia in First Remission. N. Engl. J. Med..

